# Longitudinal profiles of back pain across adulthood and their relationship with childhood factors: evidence from the 1946 British birth cohort

**DOI:** 10.1097/j.pain.0000000000001143

**Published:** 2018-02-02

**Authors:** Stella G. Muthuri, Diana Kuh, Rachel Cooper

**Affiliations:** MRC Unit for Lifelong Health and Ageing at UCL, London, United Kingdom

**Keywords:** Back pain, Long term, Birth cohort, Childhood, Risk factors, Latent class analysis

## Abstract

This study aimed to (1) characterise long-term profiles of back pain across adulthood and (2) examine whether childhood risk factors were associated with these profiles, using data from 3271 participants in the Medical Research Council National Survey of Health and Development. A longitudinal latent class analysis was conducted on binary outcomes of back pain at ages 31, 36, 43, 53, 60 to 64, and 68 years. Multinomial logistic regression models were used to examine associations between selected childhood risk factors and class membership; adjusted for sex, adult body size, health status and behaviours, socioeconomic position, and family history of back pain. Four profiles of back pain were identified: no or occasional pain (57.7%), early-adulthood only (16.1%), mid-adulthood onset (16.9%), and persistent (9.4%). The “no or occasional” profile was treated as the referent category in subsequent analyses. After adjustment, taller height at age 7 years was associated with a higher likelihood of early-adulthood only (relative risk ratio per 1 SD increase in height = 1.31 [95% confidence interval: 1.05-1.65]) and persistent pain (relative risk ratio = 1.33 [95% confidence interval: 1.01-1.74]) in women (*P* for sex interaction = 0.01). Factors associated with an increased risk of persistent pain in both sexes were abdominal pain, poorest care in childhood, and poorer maternal health. Abdominal pain and poorest housing quality were also associated with an increased likelihood of mid-adulthood onset pain. These findings suggest that there are different long-term profiles of back pain, each of which is associated with different early life risk factors. This highlights the potential importance of early life interventions for the prevention and management of back pain.

## Abstract 2

Supplemental Digital Content is Available in the Text.

In a British birth cohort study, 4 distinct long-term back pain profiles were identified, each of which was associated with different early life risk factors.

## 1. Introduction

Back pain is one of the most commonly reported health problems worldwide and a leading cause of years lived with disability.^26^ It is a major cause of activity limitation and work absences^38,46^ and imposes a major socioeconomic burden to both the individual and society^2,21^ with recent estimates suggesting that low back and neck pain account for a significant proportion of health care spending globally.^2,21^

Previous systematic reviews on the prevalence of back pain have documented increases across earlier adulthood with some evidence of decline after middle age.^10,24^ However, it has been suggested that older people may have been underrepresented in these studies, and other studies report that back pain remains one of the most commonly reported symptoms in later life^14,39,48^ and that the prevalence of severe or disabling back pain increases at older ages.^3,4,55^

Back pain is often a recurrent intermittent problem with prior history predictive of future episodes.^7,25^ Therefore, it has been recommended that the course of back pain across life should be considered.^7^ This would not only help to identify and characterise groups of individuals with differing experience of back pain over time but also aid in the development of appropriate interventions that could prevent or alleviate burdens that may arise from back pain in later adulthood. However, the long-term profiles of back pain in the general adult population have not yet been well characterised. While there are existing longitudinal studies describing the course of back pain across adulthood as recovering, fluctuating, persistent, or recurring,^29–31,54,56,57,59^ most of these have sampled populations from specific settings, for example, workplace^29^ or clinical^31^ or have excluded those without back pain at baseline.^54,57^ They also tend to have short follow-up periods (eg, 1-5 years)^30,54,57,59^ or limited follow-up points (eg, 3 repeat measurements).^29,56^ Consequently, the profiles described are unlikely to reflect the long-term course of back pain in the general adult population.

The aetiology of pain is underpinned by complex dynamic interactions between biological, psychological, and social factors acting on a range of different pathways across life.^11^ This has led to recommendations to apply a life course approach to back pain based on the biopsychosocial model.^6,11^ In support of this, there is a growing literature demonstrating that early exposure to repeated and prolonged stress or pain may alter subsequent physiological and behavioural responsiveness to stressors.^13,16^ Epidemiological research has also shown that back pain in adulthood may have its origins in earlier life with a range of different factors implicated.^7,27^ However, these studies have all considered back pain at a single time point in adulthood, and so, none has examined whether childhood factors are associated with different long-term profiles of back pain across adulthood.

Using data from the Medical Research Council (MRC) National Survey of Health and Development (NSHD), we aimed to (1) use latent class analysis (LCA) to identify profiles of back pain over the adult life course and (2) examine whether selected factors in childhood are associated with these profiles, taking account of known adult risk factors.

## 2. Methods

### 2.1. Study participants

The MRC NSHD is a population sample of 5362 single, legitimate births that occurred in England, Wales, and Scotland in 1 week of March 1946. Participants have been prospectively followed from birth with data collections conducted at least every 2 years during childhood and at 5- to 10-year intervals in adulthood. Study participation rates have remained high across life.^35,36,52,58^ The 24th data collection was conducted between 2014 and 2015 when study members were aged 68 to 69 years. Of the original study sample, 2816 (53%) were still living in England, Scotland, and Wales. Of these, 2370 (84.2%) completed a postal questionnaire, the majority within 2 months of receipt. In addition, a postal questionnaire was sent to 126 study members living abroad who remain in contact with the study of whom 83 (65.9%) returned a questionnaire. Of the remaining 2420 study members, 957 (17.8%) had already died, 620 (11.6%) had previously withdrawn permanently, 448 (8.3%) had emigrated and were no longer in contact with the study, and 395 (7%) had remained untraceable for more than 5 years.^36^ All waves of data collection have complied with ethical standards. Ethical approval for the data collection at age 68 to 69 years was obtained from the Queen Square Research Ethics Committee (14/LO/1073) and the Scotland A Research Ethics Committee (14/SS/1009), and written informed consent was obtained from study members.

### 2.2. Assessment of back pain

Data on back pain have been ascertained during each of the 6 most recent waves of assessment, using postal questionnaires at ages 31 and 68 years, and during nurse interviews at ages 36, 43, 53, and 60 to 64 years. At each age, information provided was used to derive a binary variable indicating whether back pain was reported at that age or not. At age 31 years, participants were asked whether they had ever had trouble with their back; at ages 36, 43, 53, and 60 to 64 years, they were asked whether they had sciatica, lumbago, or recurring/severe backache all or most of the time (ever at ages 36 and 43 years and in the previous 12 months at ages 53 and 60-64 years). At age 68 years, participants were asked whether they had experienced any ache or pain in the previous month, which had lasted for 1 day or longer, not including pain occurring during the course of a feverish illness such as flu. Those who responded positively were asked to shade the location of their pain using a 4-view body manikin. We then classified those who shaded any back site as having back pain, whereas those who did not report pain at any back site were coded as no back pain.

### 2.3. Childhood risk factors

Childhood risk factors were identified a priori based on the biopsychosocial model of chronic pain,^11^ which included factors examined in previous studies.^7,27^ These factors were then grouped into 4 specific domains as follows: anthropometrics, physical and psychological health, family environment, and socioeconomic.

#### 2.3.1. Anthropometric factors

Weight (in kilograms) and height (in centimetres) were measured by nurses using standardised protocols at age 7 years, and body mass index (BMI) (weight [kg]/height [m^2^]) was calculated. To facilitate comparisons, height and BMI were sex standardised to a mean value of 0 and an SD of 1.

#### 2.3.2. Physical and psychological health

Information on illnesses and injury during early life, including hospital admission, age at admission, diagnosis, name of hospital, and length of hospital stay was obtained from mothers' reports across childhood, and serious illness was defined as any experience of physical illness before the age of 16, which required hospital admission of at least 28 days.^45^

Abdominal pain over the previous year at age 15 years, previously found to be related to unexplained multiple physical symptoms in adulthood,^23^ was also considered and was based on mothers' reports at this age.

At ages 13 and 15 years, teachers were asked to rate the behaviour of the study member relative to other children in the classroom using the Rutter Child Behaviour Questionnaire (Rutter B Scale).^51^ Previous work has used exploratory factor analysis of these ratings to identify conduct and emotional problems, coded as absent, mild, or severe.^60^

#### 2.3.3. Family environment

At age 4 years, a health visitor was asked to report on the care of the study member and cleanliness of their home. Based on these reports, a score for care of house and child was derived by allocating 1 point for each of very clean house, very clean child, at least adequate shoes, at least adequate clothes, and the mother coped well when caring for the child. This score was then categorized into 3 groups of similar size: best, intermediate, and worst.^34^

Information on family disruption due to parental divorce before the study member was age 15 years came from mothers' reports during childhood and from study participants' reports when they were age 26 years. Also at age 15 years, mothers were asked to rate their own general health and that of their husband's on a 5-point scale ranging from “excellent” to “bad.”

#### 2.3.4. Socioeconomic factors

Parental levels of education were obtained from mothers' reports when participants were aged 6 years and distinguished those with secondary education or higher from those with only a primary education or none. Parental occupational class was assessed using father's occupation when the participant was 4 years old (where this was unknown, father's occupation at age 11 or 15 years was used) and classified as nonmanual (I or II or III nonmanual) or manual (III manual, IV, or V). Information on material home conditions (including type, age, tenure and state of repair of the dwelling, and crowding in the household) was collected during mothers' interviews when the study member was age 4 years. A score for house quality was then derived by allocating 1 point for each of dwelling in very good repair, dwelling built since 1919, and no overcrowding (<1.5 people per room), and further categorised into best, intermediate, and worst.^34^

### 2.4. Covariates

Covariates from early adulthood were selected a priori from different domains of the biopsychosocial model^11^ including those that have been identified in previous studies as adult risk factors of back pain.^20,25^

Weight (kilogram) and height (meter) were measured at age 36 years by a trained nurse during a clinical assessment at this age, and BMI was calculated as weight (kg)/height (m^2^).

A summary measure of psychiatric disorders experienced between ages 15 and 32 years was created using information from various sources (psychiatric hospital admission, outpatient psychiatric care after the age of 15, antipsychotic drug use, correspondence with general practitioners, and study members' during routine interviews) between these 2 ages and grouped into severe (any hospital admission), minor, or no disorder.

The highest education level achieved by age 26 years was grouped into no qualifications, up to O-level or equivalent (secondary-level qualification usually attained at age 16 years), or A-level (advanced secondary-level education usually attained at age 18 years), or equivalent and above. Own occupation at age 26 years was categorised according to Registrar General's social classification into 3 groups: nonmanual (I, II, and IIINM); manual (IIIM, IV, and V); and not employed.

Smoking status was assessed by self-report from age 16 years up to age 36 years and categorised as never, ex-, and current smoker.

At ages 36 and 43 years, study members were asked about parental experience of back pain. If during either of these assessments they reported that at least one of their parents had experienced back pain, this was coded as family history of back pain (yes vs no).

### 2.5. Statistical analysis

Data analyses were conducted in 2 steps. First, to identify profiles of back pain across adulthood (aim 1), binary outcomes of back pain at ages 31, 36, 43, 53, 60 to 64, and 68 years were modelled using longitudinal LCA. A series of models with increasing numbers of classes were fitted. Analyses included participants with at least 3 assessments of back pain (n = 3271, men = 49.8%). For individuals who did not have all 6 assessments of back pain (720 had 5, 612 had 4, and 432 had 3), full information maximum likelihood was used to handle missing values under a missing at random assumption.^40^ We also compared LCA models that allowed men and women to differ (variant) with those that constrained the parameters to be the same in both sexes (invariant). In a sensitivity analysis, we repeated LCA in just those with data from all 6 assessments (n = 1507).

The most parsimonious model (ie, fewest classes) was selected using different indices of goodness of fit including the likelihood ratio χ^2^; Pearson χ^2^ test; bootstrap *P* value for likelihood ratio test; Akaike information criterion, Bayesian information Lo–Mendell–Rubin likelihood ratio test; high entropy; and meaningful interpretation of back pain profiles.^12^ All LCA were performed with Mplus version 6.12. Estimated posterior class membership probabilities and class membership from the optimal model were exported to Stata version 14.1 for subsequent analyses.

Second, multinomial logistic regression models were used to examine associations between childhood risk factors and class membership (aim 2). Initial sex-adjusted models examined the association between each potential childhood risk factor separately. Formal tests of sex interaction were performed, and where evident, sex-stratified analyses were presented. Deviation from linearity was also assessed, where appropriate, by including quadratic terms. Potential risk factors which remained significantly associated with any class membership at the 10% level in sex-adjusted models were then grouped into the 4 prespecified domains (anthropometric, physical and psychological health, family environment, and socioeconomic position). Childhood risk factors within each domain were mutually adjusted for each other, and those that remained associated with any class membership were then included in an additional model that included adjustment for adult covariates. Childhood factors that remained associated with any outcome in domain-specific models were then selected for inclusion in a final model that combined childhood factors from all domains. All models included the maximum number of participants with valid data on childhood factors and class membership. To reduce potential data loss, adult covariates with missing values (ie, BMI [n = 256], height [n = 249], psychiatric symptoms in early adulthood [n = 63], educational attainment [n = 167], occupational class [n = 274], smoking status [n = 226], and family history of back pain [n = 53] were imputed using multiple imputation by chained equations.^53^ Analyses were performed across 20 imputed datasets and combined using Rubin rules.

## 3. Results

**Figure 1.**
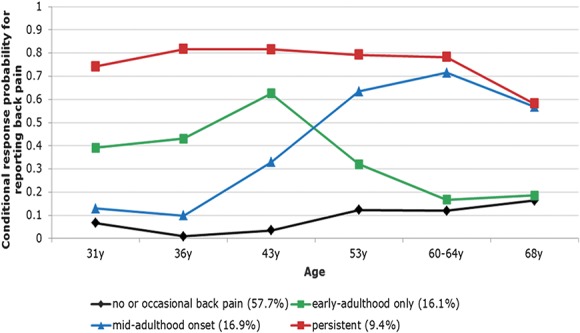
Four longitudinal classes of back pain from age 31 to 68 years, n = 3271.

A 4-class LCA model was identified as the best fit for describing distinct back pain profiles across adulthood (Table 1). Figure 1 illustrates results for the 4-class LCA model, which reflects the following patterns: a group with a high probability of reporting no or occasional back pain across adulthood (“no or occasional,” n = 1888 [57.7%]); a group with a higher probability of reporting back pain earlier in adulthood but with a lower probability of back pain at later ages (“early-adulthood only,” n = 525 [16.1%]); a group with an increasing probability of reporting back pain from age 36 years onwards (“mid-adulthood onset,” n = 552 [16.9%]); and a group with a higher probability of reporting back pain at all ages in adulthood (“persistent,” n = 306 [9.4%]). These back pain profiles were similar to those observed in sex-specific latent classes (Figure S1, Supplemental Digital Content 1, available online at http://links.lww.com/PAIN/A523), but the model fit was best in the combined model (Table S1, Supplemental Digital Content 1, online at http://links.lww.com/PAIN/A523); therefore, it was used in subsequent analyses.

**Table 1:**
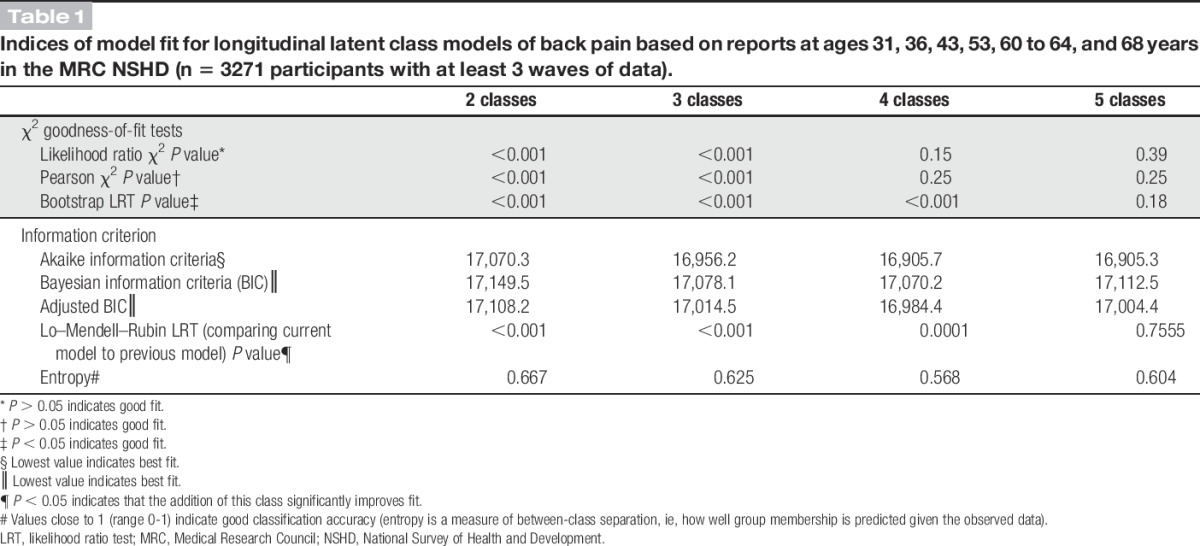
Indices of model fit for longitudinal latent class models of back pain based on reports at ages 31, 36, 43, 53, 60 to 64, and 68 years in the MRC NSHD (n = 3271 participants with at least 3 waves of data).

Table 2 presents the characteristics of the study participants. A higher proportion of men than women were classified as having no or occasional back pain across adulthood (61% vs 55%), *P* = 0.001. In terms of childhood factors, males were more likely than females to be taller and heavier at age 7 years, to have had conduct problems, and to have lived in poor-quality housing but less likely to have had emotional problems or abdominal pain during adolescence.

**Table 2:**
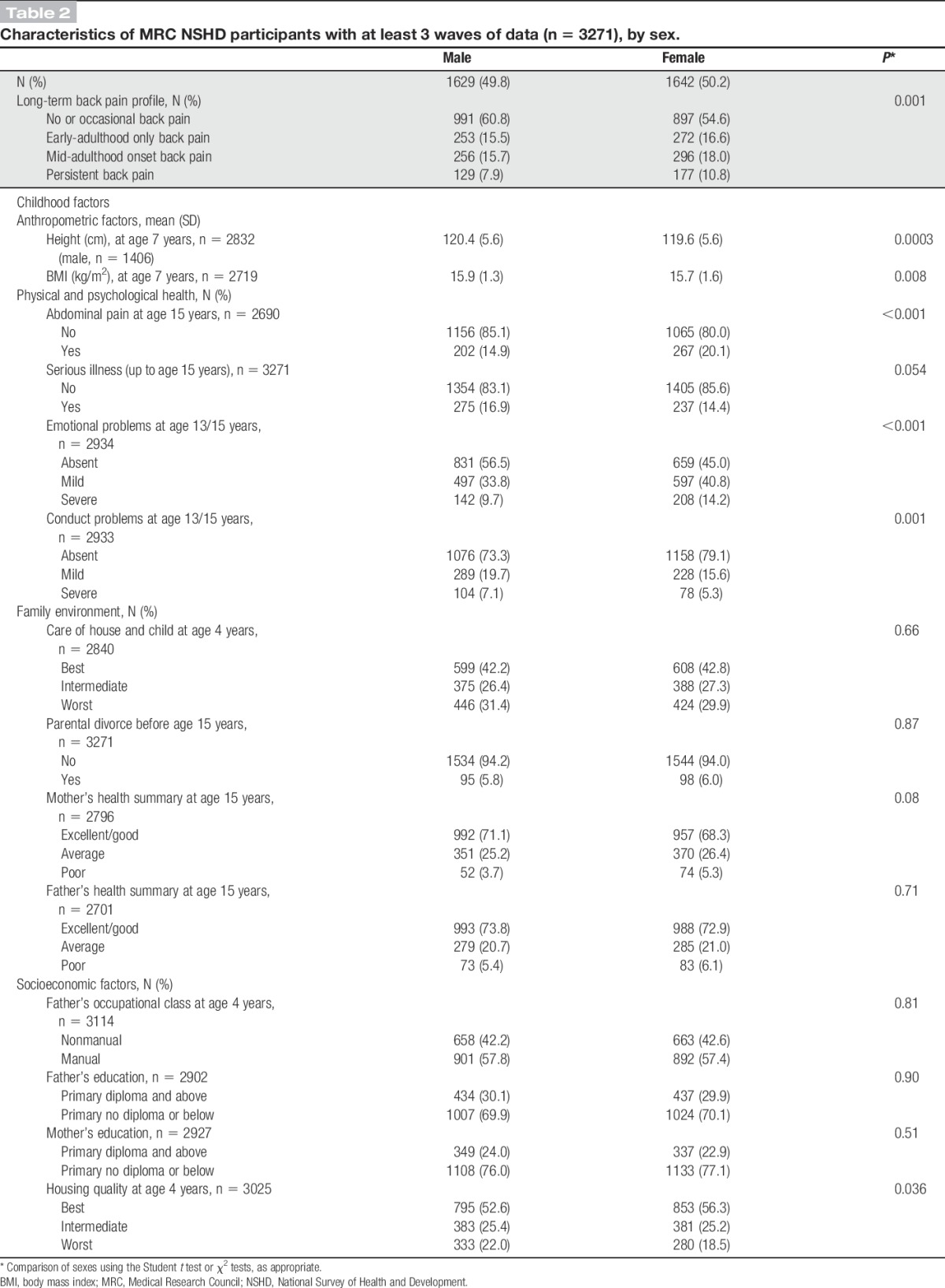
Characteristics of MRC NSHD participants with at least 3 waves of data (n = 3271), by sex.

### 3.1. Associations of childhood risk factors with long-term back pain profiles

#### 3.1.1. Anthropometric factors

There were sex differences in the associations between childhood height and back pain profiles (*P* for sex interaction = 0.014). Women who were taller at age 7 years had a higher likelihood of early-adulthood only {relative risk ratio [RRR] per 1 SD increase in height = 1.37 (95% confidence interval [CI]: 1.18-1.59)} and persistent back pain (RRR = 1.48 [95% CI: 1.24-1.76]) when compared with women in the no or occasional back pain profile (Table 3). No associations were observed between childhood height and back pain profiles in men. Greater childhood BMI was also associated with a higher likelihood of persistent pain (RRR = 1.16 [95% CI: 1.02-1.32]) after adjustment for sex (Table 3). These associations were maintained after mutual adjustment (Table 4, model 1), but after adjustment for adult covariates, only childhood height remained independently associated with early-adulthood and persistent back pain in women (Table 4, model 2).

**Table 3:**
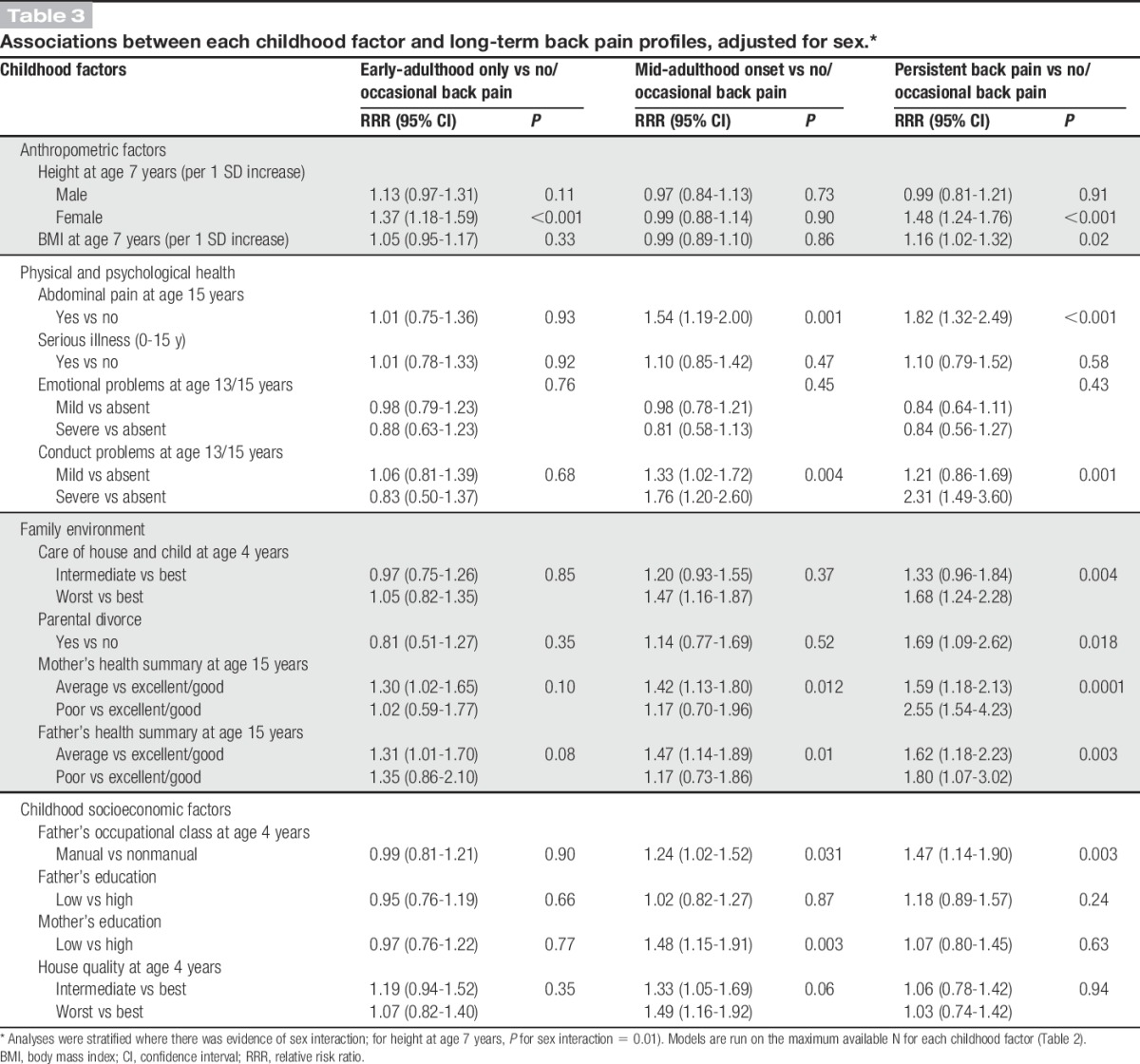
Associations between each childhood factor and long-term back pain profiles, adjusted for sex.*

**Table 4:**
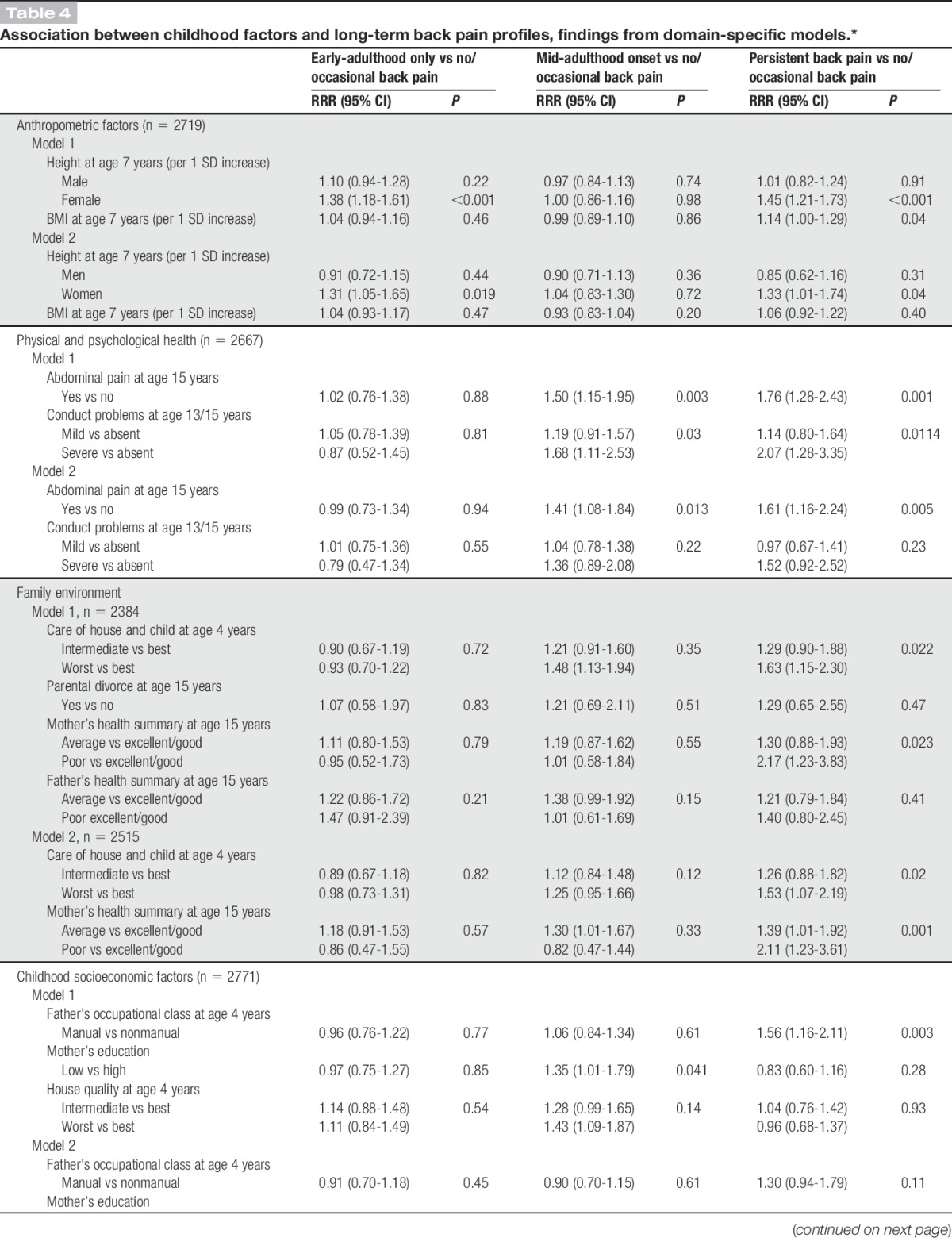

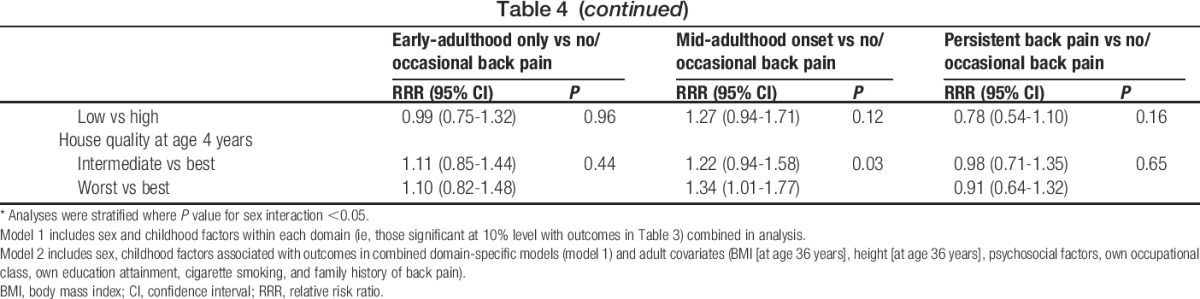
Association between childhood factors and long-term back pain profiles, findings from domain-specific models.*

#### 3.1.2. Physical and psychosocial health

Abdominal pain and conduct problems were associated with a higher likelihood of mid-adulthood onset back pain and persistent back pain, after adjusting for sex (Table 3). No associations were found with serious illness in childhood or emotional problems. After mutual adjustment, the RRRs of pain for abdominal pain and conduct problems were partly attenuated (Table 4, model 1). After further adjustment for adult covariates, abdominal pain remained associated with mid-adulthood onset pain and persistent back pain, whereas the positive association of conduct problems was fully attenuated (Table 4, model 2).

#### 3.1.3. Family environment

In sex-adjusted models, poorer care of house and child, parental divorce, and poorer maternal and paternal health were associated with an increased likelihood of persistent back pain, whereas only poorer paternal health was associated with mid-adulthood onset pain (Table 3). After mutual adjustment of these factors, worst care of house and child and poorer maternal health remained associated with persistent back pain (Table 4, model 1); these associations remained even after additional adjustment for adult covariates (Table 4, model 2).

#### 3.1.4. Socioeconomic factors

Father's manual occupational class, low maternal educational attainment, and poorer housing quality were associated with mid-adulthood onset and persistent back pain profiles after adjustment for sex (Table 3). These associations were maintained after mutual adjustment (Table 4, model 1), but after adjustment for adult covariates, only poor housing quality remained associated with an increased likelihood of mid-adulthood onset back pain (RRR = 1.34 [95% CI: 1.01-1.77]) (Table 4, model 2).

### 3.2. Associations of back pain profiles with selected childhood factors—mutually adjusted models

Five childhood factors, namely, height at age 7 years, abdominal pain, care of house and child, maternal health, and housing quality, remained associated with pain profiles in domain-specific multivariable models after adjustment for adult covariates (Table 4, model 2). These were the factors that were simultaneously adjusted for in addition to adult covariates in a subsequent model (Table 5). Childhood height (in women only), poorer care of house and child, and poorer maternal health remained associated with an increased likelihood of persistent back pain in fully adjusted models, whereas the positive association with abdominal pain was fully attenuated (Table 5, model 2). The association between abdominal pain and mid-adulthood onset back pain was maintained. However, poorer housing quality was no longer associated with any back pain profile when factors from all domains were examined together (Table 5).

**Table 5:**
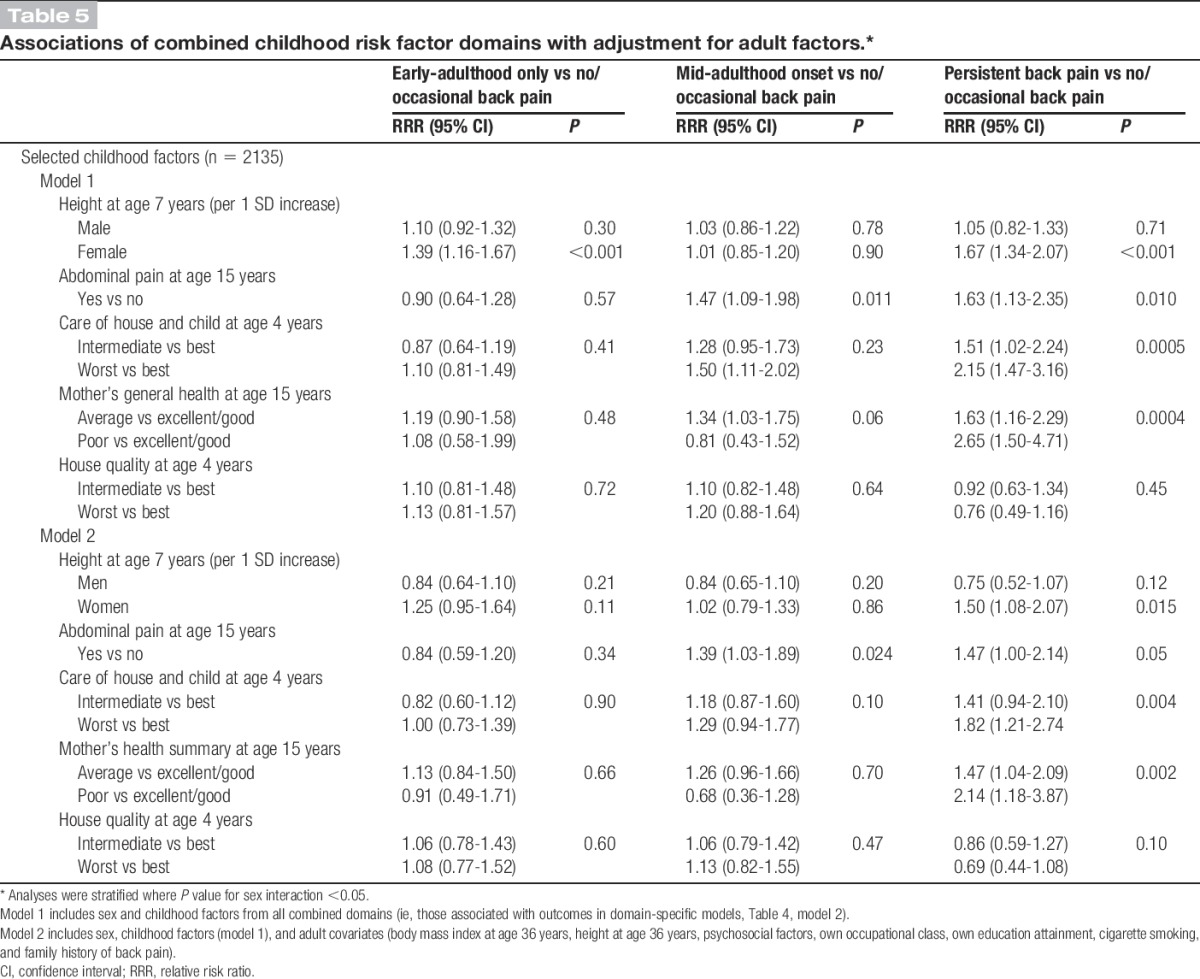
Associations of combined childhood risk factor domains with adjustment for adult factors.*

## 4. Discussion

Our study has identified subgroups of individuals in the general population with differing profiles of back pain across adulthood, which were related to different childhood risk factors. After adjustment for other childhood and adulthood factors, taller childhood height (women only), abdominal pain in adolescence, lower care of house and child, and poorer maternal health were associated with an increased likelihood of persistent back pain; abdominal pain in adolescence was associated with a higher likelihood of “mid-adulthood” onset back pain; and greater childhood height was associated with “early-adulthood” back pain among women.

### 4.1. Comparison of findings with previous studies

Our findings build on those from previous studies that have characterised the course of back pain in the general adult population.^19,54,56,59^ The 4 distinct long-term profiles of back pain identified have some similarities with those previously described.^19,54,56,59^ For example, our finding that 9% of the population had “persistent back pain” is consistent with estimates in some other studies.^19,54,56,59^ We also identified 2 novel back pain profiles—early-adulthood only and mid-adulthood onset. The long follow-up period and multiple repeat back pain assessments may have contributed to the identification of these subgroups. Other differences between the characteristics of identified back pain profiles in our study when compared with others may reflect cohort differences, differences in case definitions of back pain, or the analytical method used to characterise long-term back pain profiles.

Our study goes beyond these population-based studies that have characterised long-term back pain profiles and extends previous studies that have examined associations between several childhood risk factors and back pain at a single age in adulthood.^18,32,33,41,44,50^ A number of childhood factors including anthropometric (height^1,17^); physical and psychosocial health (serious illness,^32^ abdominal pain,^28^ conduct problems, or emotional problems^28,44^); family environment (poor paternal health^18^); and socioeconomic factors (parental educational attainment^33,44^ and parental unemployment^32^) have been shown to be associated with back pain in adolescence and adulthood. Our study has considered long-term back pain profiles across adulthood and found that several childhood factors were associated with these. Our findings also highlight that the associations of childhood factors with adult back pain are potentially mediated by differential exposure to adult risk factors. Specifically, adjustment for adult body size (ie, height and BMI), health status and behaviours (ie, psychological distress and smoking), socioeconomic position, and family history of back pain attenuated associations of conduct problems in adolescence and childhood socioeconomic position with back pain profiles. The findings are consistent with those of previous studies, which have shown no associations of low childhood socioeconomic indicators and emotional or conduct behaviours with back pain in adulthood after adjustment for other factors in childhood and early adulthood.^41,44,50^

### 4.2. Potential explanations of the findings

Long-term persistent back pain across adulthood was the least common profile in our study population and was associated with multiple childhood risk factors including taller height at age 7 years (in women only), abdominal pain in adolescence, lower care of house and child, and poorer maternal health. Thus, it may be that for the same group of individuals who have experienced back pain across adulthood consistently, several potential mechanisms acting throughout life may be involved.

Identification of early-adulthood only and mid-adulthood onset back pain profiles suggests that midlife may be a transition period when some people begin to recover from back pain, whereas others begin to experience symptoms that persist. Our findings further demonstrate that different childhood risk factors may be associated with early-adulthood and mid-adulthood onset; thus, it is likely that these back pain profiles may reflect different distinct underlying aetiologies. For example, greater childhood height was associated with an increased likelihood of early-adulthood only in women, suggesting that this pain profile may be explained by physical factors, whereas associations of abdominal pain in adolescence with the mid-adulthood onset back pain profile suggest that childhood psychosocial factors may be involved. Previous observational studies also suggest that vulnerability to back pain in midlife may be explained by a combination of adult health status and behaviours and socioeconomic factors.^25^ As degenerative back disorders become increasingly common in people aged 40 years and older,^15^ it is likely that the mid-adulthood onset profile includes individuals with specific and nonspecific back pain. However, the early-adulthood only subgroup may be similar to the “recovering” group identified in primary care patient populations,^5,8^ perhaps suggesting that some of those who experience back pain in earlier adulthood including those who may not necessarily seek beneficial treatment may see a natural improvement in their symptoms.

Previous longitudinal studies have also shown positive associations between adult height and back pain; however, no significant sex differences were established.^20,37^ In multivariable analyses, we found positive associations of adult height with early-adulthood and persistent back pain, but associations were not significantly different in men and women (data not shown). Thus, it may be that associations observed with childhood height in women only may reflect sex differences in growth patterns during childhood.^49^ In post hoc analysis, we found that girls had achieved a significantly higher percentage of adult height (PAH) by age 7 years (mean = 73.7%, SD = 2.4) than boys (mean = 68.6%, SD = 2.2), and 1 unit increase in PAH was associated with an increased likelihood of both early-adulthood and persistent back pain in women but not in men (results not shown). By contrast, previous work in the NSHD reported no associations between PAH (analysed as quintiles) attained at different ages in childhood and a history of back pain at age 43 years.^37^ The apparent inconsistencies of the findings may be due to differences in the definition of the outcome. It is also possible that other aspects of development in early life such as the length of a child's trunk^9,47^ may be important in determining susceptibility to back injuries or back disorders and warrants future investigation in adult populations. Further research to investigate the associations of rates of growth in childhood with adult back pain may also help to explain this finding.

We found strong associations of abdominal pain at age 15 years with mid-adulthood onset and persistent back pain in adulthood even after accounting for adult factors. Previous findings from the NSHD have linked abdominal pain in childhood with an increased likelihood of psychiatric disorders^22^ and unexplained multiple physical symptoms^23^ in adulthood. It has been postulated that somatic pain experiences in early life may cause irreversible change in the central pain processing mechanisms and potentially alter pain pathways,^13,16^ which may induce long-term alterations in pain sensitivity that could extend across adulthood.

The associations of poorer care of house and child and poorer maternal health with persistent back pain were likely to reflect conditions that characterised the early home environment. They have also been associated with lower physical health-related quality of life, limited physical functioning^43^ and premature mortality^34^ in previous NSHD analyses. It has been suggested that a complex interplay may exist between biological, genetic, and environmental conditions, which may, in turn, influence the nurturing qualities of physical and social environments where children live that are likely to shape their long-term health.^42^

### 4.3. Methodological considerations

A key strength of our study is the large population-based sample of adults, with repeat back pain measurements collected at 6 time points over 37 years of follow-up. The prospective nature of the study also enabled examination of a wide range of factors across childhood.

Our study has several limitations. First, back pain was not assessed in the same way across adulthood. However, the longitudinal LCA approach used was appropriate for summarising repeat binary back pain outcomes. We also identified 4 distinct long-term profiles of back pain that had face and internal validity. The assignment probabilities and entropy values suggest that classes were well separated (Table 1). Similarly, findings from sex-specific latent classes and latent classes in those with repeat back pain data at all 6 assessments (Figure S2 and Table S2, Supplemental Digital Content 1, available online at http://links.lww.com/PAIN/A523) also confirmed 4 distinct back pain profiles that had similar proportions and characteristics as the maximum sample used in the analyses. A second limitation is that back pain outcomes focus on the presence or absence of pain at multiple time points across adulthood. Thus, it is possible that a cohort with a case definition that includes age at pain onset, frequency, duration, intensity and recurrence of pain, and pain-related disability might identify additional longitudinal profiles of back pain not captured in this study. Third, we took a 2-stage approach in our analyses; typologies of back pain across adulthood were identified, and then, individual childhood risk factors were related to derived class memberships. This approach did not correct for the uncertainty in class membership by weighting models using estimated posterior probabilities that may have resulted in an overestimation of associations. Fourth, it is possible that inclusion of adult covariates such as body size and socioeconomic factors, which may be on the causal pathway, could be viewed as an overadjustment. In addition, there are some other factors, such as physical activity participation in childhood and adolescence, which may be important in their own right and could potentially confound the observed associations which it was not possible to take into account as they were not measured until adulthood in the NSHD. Finally, as with all longitudinal studies, there has also been attrition over time, although NSHD participation rates are high^36^ and the study sample remains broadly representative of the British born population.^52^

## 5. Conclusions

In this large British birth cohort study, we identified 4 distinct long-term profiles of back pain and found that each profile was associated with different early life risk factors, even after adjusting for known adulthood risk factors. These findings improve our understanding of the long-term course of back pain in the general population and highlight the potential importance of early life interventions for its prevention and management.

## Conflict of interest statement

The authors have no conflict of interest to declare.

This work is supported by the United Kingdom Medical Research Council, which provides core funding for the MRC National Survey of Health and Development and supports S.G.M., D.K., and R.C. (programme codes: MC_UU_12019/1 and MC_UU_12019/4). S.G.M. is also supported by MRC grant MR/L010399/1.

Preliminary findings from this work were presented at the Annual Scientific Meeting of the Society for Social Medicine, Manchester, United Kingdom, September 2017.

Data used in this publication are available to bona fide researchers upon request to the NSHD Data Sharing Committee via a standard application procedure. Further details can be found at http://www.nshd.mrc.ac.uk/data. doi: 10.5522/NSHD/Q101; doi: 10.5522/NSHD/Q102; doi: 10.5522/NSHD/Q103.

## Appendix A. Supplemental digital content

Supplemental digital content associated with this article can be found online at http://links.lww.com/PAIN/A523.

## References

[1] Balagué F Troussier B Salminen JJ Non-specific low back pain in children and adolescents: risk factors. Eur Spine J 1999;8:429–38. 10664299 10.1007/s005860050201 PMC3611213

[2] Dieleman JL Baral R Birger M US spending on personal health care and public health, 1996-2013. JAMA 2016;316:2627–46. 28027366 10.1001/jama.2016.16885 PMC5551483

[3] Dionne CE Dunn KM Croft PR Does back pain prevalence really decrease with increasing age? A systematic review. Age Ageing 2006;35:229–34. 16547119 10.1093/ageing/afj055

[4] Docking RE Fleming J Brayne C Zhao J Macfarlane GJ Jones GT Epidemiology of back pain in older adults: prevalence and risk factors for back pain onset. Rheumatology 2011;50:1645–53. 21606130 10.1093/rheumatology/ker175

[5] Downie AS Hancock MJ Rzewuska M Williams CM Lin C-WC Maher CG Trajectories of acute low back pain: a latent class growth analysis. PAIN 2016;157:225–34. 26397929 10.1097/j.pain.0000000000000351

[6] Dunn KM Extending conceptual frameworks: life course epidemiology for the study of back pain. BMC Musculoskelet Disord 2010;11:23. 20122264 10.1186/1471-2474-11-23 PMC2829505

[7] Dunn KM Hestbaek L Cassidy JD Low back pain across the life course. Best Pract Res Clin Rheumatol 2013;27:591–600. 24315141 10.1016/j.berh.2013.09.007

[8] Dunn KM Jordan K Croft PR Characterizing the course of low back pain: a latent class analysis. Am J Epidemiol 2006;163:754–61. 16495468 10.1093/aje/kwj100

[9] Fairbank JCT Pynsent PB Van Poortvliet JA Phillips H Influence of anthropometric factors and joint laxity in the incidence of adolescent back pain. Spine (Phila Pa 1976) 1984;9:461–4. 6238421 10.1097/00007632-198407000-00007

[10] Fejer R Leboeuf-Yde C Does back and neck pain become more common as you get older? A systematic literature review. Chiropr Man Therap 2012;20:24. 10.1186/2045-709X-20-24 PMC3526387 22883425

[11] Gatchel RJ Peng YB Peters ML Fuchs PN Turk DC The biopsychosocial approach to chronic pain: scientific advances and future directions. Psychol Bull 2007;133:581–624. 17592957 10.1037/0033-2909.133.4.581

[12] Geiser C Data analysis with mplus. New York City, NY: The Guilford Press, 2013.

[13] Grunau RE Holsti L Haley DW Oberlander T Weinberg J Solimano A Whitfield MF Fitzgerald C Yu W Neonatal procedural pain exposure predicts lower cortisol and behavioral reactivity in preterm infants in the NICU. PAIN 2005;113:293–300. 15661436 10.1016/j.pain.2004.10.020 PMC1447527

[14] Hartvigsen J Christensen K Pain in the back and neck are with us until the end: a nationwide interview-based survey of Danish 100-year-olds. Spine (Phila Pa 1976) 2008;33:909–13. 18404112 10.1097/BRS.0b013e31816b45f1

[15] Hartvigsen J Christensen K Frederiksen H Back pain remains a common symptom in old age. A population-based study of 4486 Danish twins aged 70-102. Eur Spine J 2003;12:528–34. 12748896 10.1007/s00586-003-0542-y PMC3468008

[16] Hermann C Hohmeister J Demirakça S Zohsel K Flor H Long-term alteration of pain sensitivity in school-aged children with early pain experiences. PAIN 2006;125:278–85. 17011707 10.1016/j.pain.2006.08.026

[17] Hershkovich O Friedlander A Gordon B Arzi H Derazne E Tzur D Shamis A Afek A Associations of body mass index and body height with low back pain in 829,791 adolescents. Am J Epidemiol 2013;178:603–9. 23690249 10.1093/aje/kwt019

[18] Hestbaek L Korsholm L Leboeuf-Yde C Kyvik KO Does socioeconomic status in adolescence predict low back pain in adulthood? A repeated cross-sectional study of 4,771 Danish adolescents. Eur Spine J 2008;17:1727. 18830719 10.1007/s00586-008-0796-5 PMC2587673

[19] Hestbaek L Leboeuf-Yde C Engberg M Lauritzen T Bruun NH Manniche C The course of low back pain in a general population. results from a 5-year prospective study. J Manipulative Physiol Ther 2003;26:213–19. 12750654 10.1016/s0161-4754(03)00006-x

[20] Heuch I Heuch I Hagen K Zwart JA Association between body height and chronic low back pain: a follow-up in the Nord-Trøndelag Health Study. BMJ Open 2015;5:e006983. 10.1136/bmjopen-2014-006983 PMC4480023 26078308

[21] Hong J Reed C Novick D Happich M Costs associated with treatment of chronic low back pain: an analysis of the UK general practice research database. Spine (Phila Pa 1976) 2013;38:75–82. 23038621 10.1097/BRS.0b013e318276450f

[22] Hotopf M Carr S Mayou R Wadsworth M Wessely S Why do children have chronic abdominal pain, and what happens to them when they grow up? Population based cohort study. BMJ 1998;316:1196–200. 9552994 10.1136/bmj.316.7139.1196 PMC28520

[23] Hotopf M Mayou R Wadsworth M Wessely S Childhood risk factors for adults with medically unexplained symptoms: results from a national birth cohort study. Am J Psychiatry 1999;156:1796–800. 10553745 10.1176/ajp.156.11.1796

[24] Hoy D Bain C Williams G March L Brooks P Blyth F Woolf A Vos T Buchbinder R A systematic review of the global prevalence of low back pain. Arthritis Rheum 2012;64:2028–37. 22231424 10.1002/art.34347

[25] Hoy D Brooks P Blyth F Buchbinder R The Epidemiology of low back pain. Best Pract Res Clin Rheumatol 2010;24:769–81. 21665125 10.1016/j.berh.2010.10.002

[26] Hoy D March L Brooks P Blyth F Woolf A Bain C Williams G Smith E Vos T Barendregt J Murray C Burstein R Buchbinder R The global burden of low back pain: estimates from the Global Burden of Disease 2010 study. Ann Rheum Dis 2014;73:968–74. 24665116 10.1136/annrheumdis-2013-204428

[27] Jones GT Macfarlane GJ Epidemiology of low back pain in children and adolescents. Arch Dis Child 2005;90:312–16. 15723927 10.1136/adc.2004.056812 PMC1720304

[28] Jones GT Watson KD Silman AJ Symmons DPM Macfarlane GJ Predictors of low back pain in British schoolchildren: a population-based prospective cohort study. Pediatrics 2003;111:822–8. 12671119 10.1542/peds.111.4.822

[29] Kääriä S Luukkonen R Riihimäki H Kirjonen J Leino-Arjas P Persistence of low back pain reporting among a cohort of employees in a metal corporation: a study with 5-, 10-, and 28-year follow-ups. PAIN 2006;120:131–7. 16360271 10.1016/j.pain.2005.10.020

[30] Kolb E Canjuga M Bauer GF Läubli T Course of back pain across 5 years: a retrospective cohort study in the general population of Switzerland. Spine (Phila Pa 1976) 2011;36:E268–73. 21270712 10.1097/BRS.0b013e3181f324b5

[31] Kongsted A Kent P Axen I Downie AS Dunn KM What have we learned from ten years of trajectory research in low back pain? BMC Musculoskelet Disord 2016;17:220. 27209166 10.1186/s12891-016-1071-2 PMC4875630

[32] Kopec JAMDP Sayre ECB Stressful experiences in childhood and chronic back pain in the general population. Clin J Pain 2005;21:478–83. 16215332 10.1097/01.ajp.0000139909.97211.e1

[33] Kristensen P Bjerkedal T Irgens LM Early life determinants of musculoskeletal sickness absence in a cohort of Norwegians born in 1967-1976. Soc Sci Med 2007;64:646–55. 17088015 10.1016/j.socscimed.2006.09.032

[34] Kuh D Hardy R Langenberg C Richards M Wadsworth MEJ Mortality in adults aged 26-54 years related to socioeconomic conditions in childhood and adulthood: post war birth cohort study. BMJ 2002;325:1076–80. 12424168 10.1136/bmj.325.7372.1076 PMC131184

[35] Kuh D Pierce M Adams J Deanfield J Ekelund U Friberg P Ghosh AK Harwood N Hughes A Macfarlane PW Mishra G Pellerin D Wong A Stephen AM Richards M Hardy R Cohort profile: updating the cohort profile for the MRC National Survey of Health and Development: a new clinic-based data collection for ageing research. Int J Epidemiol 2011;40:e1–9. 21345808 10.1093/ije/dyq231 PMC3043283

[36] Kuh D Wong A Shah I Moore A Popham M Curran P Davis D Sharma N Richards M Stafford M Hardy R Cooper R The MRC National Survey of Health and Development reaches age 70: maintaining participation at older ages in a birth cohort study. Eur J Epidemiol 2016;31:1135–47. 27995394 10.1007/s10654-016-0217-8 PMC5206260

[37] Kuh DJL Coggan D Mann S Cooper C Yusuf E Height, occupation and back pain in a national prospective study. Rheumatology 1993;32:911–16. 10.1093/rheumatology/32.10.911 8402001

[38] Leveille SG Bean J Ngo L McMullen W Guralnik JM The pathway from musculoskeletal pain to mobility difficulty in older disabled women. PAIN 2007;128:69–77. 17055167 10.1016/j.pain.2006.08.031 PMC2555988

[39] Leveille SG Fried L Guralnik JM Disabling symptoms: what do older women report? J Gen Intern Med 2002;17:766–73. 12390552 10.1046/j.1525-1497.2002.20229.x PMC1495119

[40] Little R Rubin D Statistical analysis with missing data. New York: John Wiley and Sons, Inc, 1987.

[41] Macfarlane GJ Norrie G Atherton K Power C Jones GT The influence of socioeconomic status on the reporting of regional and widespread musculoskeletal pain: results from the 1958 British Birth Cohort Study. Ann Rheum Dis 2009;68:1591–5. 18952642 10.1136/ard.2008.093088

[42] Maggi S Irwin LJ Siddiqi A Hertzman C The social determinants of early child development: an overview. J Paediatr Child Health 2010;46:627–35. 20796183 10.1111/j.1440-1754.2010.01817.x

[43] Mishra GD Black S Stafford M Cooper R Kuh D; National Survey of Health and Development Scientific and Data Collection Team. Childhood and maternal effects on physical health related quality of life five decades later: the British 1946 birth cohort. PLoS One 2014;9:e88524. 24670776 10.1371/journal.pone.0088524 PMC3966737

[44] Mustard CA Kalcevich C Frank JW Boyle M Childhood and early adult predictors of risk of incident back pain: Ontario child health study 2001 follow-up. Am J Epidemiol 2005;162:779–86. 16150891 10.1093/aje/kwi271

[45] Muthuri SG Kuh D Bendayan R Macfarlane GJ Cooper R Chronic physical illness in early life and risk of chronic widespread and regional pain at age 68: evidence from the 1946 British birth cohort. PAIN 2016;157:2382–9. 27547897 10.1097/j.pain.0000000000000663 PMC5028158

[46] Natvig B Eriksen W Bruusgaard D Low back pain as a predictor of long-term work disability. Scand J Public Health 2002;30:288–92. 12680505 10.1080/14034940210133951

[47] Nissinen M Heliövaara M Seitsamo J Alaranta H Poussa M Anthropometric measurements and the incidence of low back pain in a cohort of pubertal children. Spine (Phila Pa 1976) 1994;19:1367–70. 8066517 10.1097/00007632-199406000-00010

[48] Patel KV Guralnik JM Dansie EJ Turk DC Prevalence and impact of pain among older adults in the United States: findings from the 2011 National Health and Aging Trends Study. PAIN 2013;154:2649–57. 24287107 10.1016/j.pain.2013.07.029 PMC3843850

[49] Poussa MS Heliövaara MM Seitsamo JT Könönen MH Hurmerinta KA Nissinen MJ Anthropometric measurements and growth as predictors of low-back pain: a cohort study of children followed up from the age of 11 to 22 years. Eur Spine J 2005;14:595–8. 15789230 10.1007/s00586-004-0872-4 PMC3489232

[50] Power C Frank J Hertzman C Schierhout G Li L Predictors of low back pain onset in a prospective British study. Am J Public Health 2001;91:1671–8. 11574334 10.2105/ajph.91.10.1671 PMC1446853

[51] Rutter M A children's behaviour questionnaire for completion by teachers: preliminary findings. J Child Psychol Psychiatry 1967;8:1–11. 6033260 10.1111/j.1469-7610.1967.tb02175.x

[52] Stafford M Black S Shah I Hardy R Pierce M Richards M Wong A Kuh D Using a birth cohort to study ageing: representativeness and response rates in the National Survey of Health and Development. Eur J Ageing 2013;10:145–57. 23637643 10.1007/s10433-013-0258-8 PMC3637651

[53] Sterne JAC White IR Carlin JB Spratt M Royston P Kenward MG Wood AM Carpenter JR Multiple imputation for missing data in epidemiological and clinical research: potential and pitfalls. BMJ 2009;338:b2393. 19564179 10.1136/bmj.b2393 PMC2714692

[54] Tamcan O Mannion AF Eisenring C Horisberger B Elfering A Müller U The course of chronic and recurrent low back pain in the general population. PAIN 2010;150:451–7. 20591572 10.1016/j.pain.2010.05.019

[55] Thomas E Mottram S Peat G Wilkie R Croft P The effect of age on the onset of pain interference in a general population of older adults: prospective findings from the North Staffordshire Osteoarthritis Project (NorStOP). PAIN 2007;129:21–7. 17084980 10.1016/j.pain.2006.09.027

[56] van Oostrom SH Monique Verschuren WM de Vet HCW Picavet HSJ Ten year course of low back pain in an adult population-based cohort—the Doetinchem cohort study. Eur J Pain 2011;15:993–8. 21429779 10.1016/j.ejpain.2011.02.007

[57] Vasseljen O Woodhouse A Bjørngaard JH Leivseth L Natural course of acute neck and low back pain in the general population: the HUNT study. PAIN 2013;154:1237–44. 23664654 10.1016/j.pain.2013.03.032

[58] Wadsworth M Kuh D Richards M Hardy R Cohort profile: the 1946 national birth cohort (MRC National Survey of Health and Development). Int J Epidemiol 2006;35:49–54. 16204333 10.1093/ije/dyi201

[59] Waxman R Tennant A Helliwell P A prospective follow-up study of low back pain in the community. Spine (Phila Pa 1976) 2000;25:2085–90. 10954640 10.1097/00007632-200008150-00013

[60] Xu MK Jones PB Barnett JH Gaysina D Kuh D Croudace TJ Richards M Adolescent self-organization predicts midlife memory in a prospective birth cohort study. Psychol Aging 2013;28:958–68. 24364401 10.1037/a0033787 PMC3906799

